# Comparison of four short axis cine acquisition protocols for the evaluation of left ventricular cardiac function

**DOI:** 10.1186/1532-429X-17-S1-P61

**Published:** 2015-02-03

**Authors:** Stephanie Marchesseau, Lynette L Teo, Ping Chai, John J Totman

**Affiliations:** 1 A*STAR-NUS Clinical Imaging Research Centre, Singapore, Singapore; 2 Cardiology, NUHCS, Sinagpore, Singapore; 3 Diagnostic Imaging, NUH, Sinagpore, Singapore

## Background

Guidelines from the SCMR board try to standardize the acquisition and post processing protocols for uniform and reproducible analysis of cardiac data. However, for short axis cine acquisition, the definition of the image orientation for data acquisition is not well defined, possibly leading to variation in the evaluation of the Left Ventricular cardiac function. Having a standard definition is particularly important for multi-center and longitudinal studies. Very little work has been done to evaluate the impact of the various proposed protocols on measurement results of Left Ventricular function, which is the aim of this study.

## Methods

20 volunteers were recruited (10 males, 10 females; age range 23-69) without CMR contraindications or known cardiovascular diseases. The study was performed on a Siemens Prisma 3T MRI. Breath-hold CINE steady state free precession (SSFP) short axis sequences were acquired (8mm slice thickness with 25% gap, 1.5 mm^2^ in-plane resolution) following 4 different acquisition planes commonly used in clinical practice (see figure): (A) *Septum:* short axis slices are perpendicular to the septum with one cutting through the septum junction. (B) *LongAxis:* short axis slices are perpendicular to the long axis with one cutting through the septum junction. (C) *Left AtrioVentricular Junctions*: one plane cuts through the left atrioventricular junction. (D) *AtrioVentricularJunctions*: one plane cuts through the right and the left atrioventricular junctions. Global LV function was quantified by a trained analyst using *Segment v1.9 R3556* (http://segment.heiberg.se). Protocols were compared pair-wise using Bland Altman analysis (RPC value), Pearson's correlation and t-tests based on measurements of ejection fraction (EF), mass, and stroke volume (SV). Results are given as mean across pairs in the Table.

**Figure 1:**
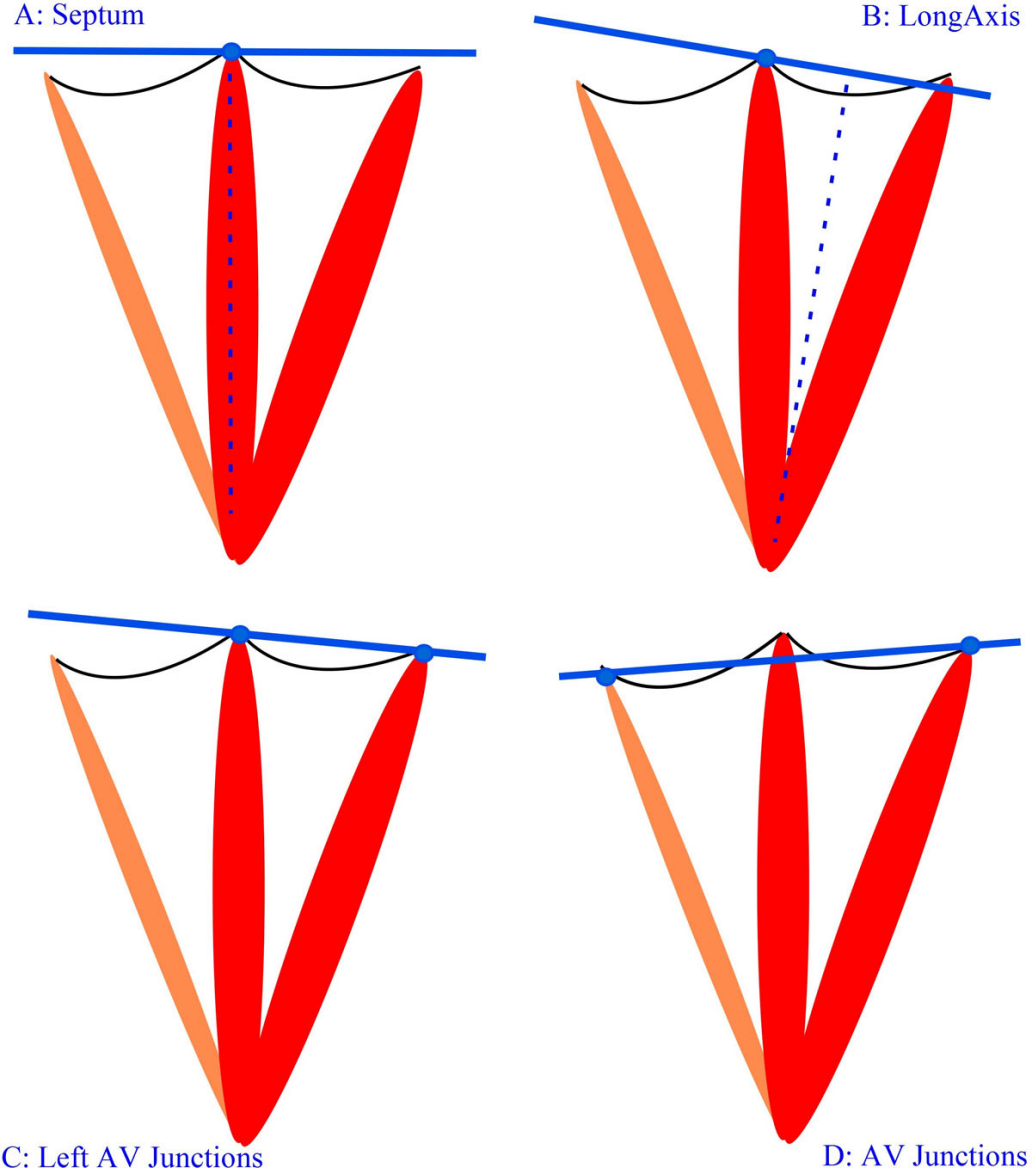
Definition of the 4 acquisition planes.

**Table 1:** Comparison of the 4 methods

	AV Junctions	Left AV Junctions	Septum	LongAxis	Mean RPC	Mean Pearson	Mean p-value
EF (%) ± std	68.50 5.20	67.60 5.80	68.50 5.20	68.20 5.20	0.04	0.92	0.38

Mass (g) ± std	132.52 32.86	135.87 34.43	134.21 33.93	132.69 32.13	17.17	0.97	0.39

SV (ml) ± std	93.49 21.29	94.99 21.37	94.59 21.80	94.01 21.75	11.50	0.96	0.53

## Results

Selection of the basal slice and the basal descent during the analysis is the first difference caused by the definition of the protocols and impacts the measurements. After analysis (see Table), the 4 protocols are highly correlated (Pearson consistently >0.9) with no protocol showing significantly different values compared with the others (RPC EF=4%, RPC Mass=17g, RPC SV=11ml; p-value consistently >0.05 with mean >0.4). Mean left ventricular EF, mass and SV are similar with every protocol.

## Conclusions

When rigorously following the SCMR guidelines for post processing, the differences in the acquisition planes do not appear to lead to significant variations in the assessment of LV function for volunteers. Consistency is preferred for clinical studies, but these results seem to indicate that a slight discrepancy in protocols would probably not affect significantly the measurement results of LV function. A similar study will be performed on a patient cohort to confirm this.

## Funding

N/A.

